# Examining Students' Motivations to Pursue a Bachelor's Degree in Nutrition

**DOI:** 10.5195/cajgh.2019.378

**Published:** 2019-05-10

**Authors:** Allison Moreno Arriaga, Rosa Dejanira Medina Terán, Cecilia Flores Martínez, María José Campos Zamora, Hilda Lissette López Lemus, Cuauhtémoc Sandoval Salazar

**Affiliations:** Department of Nursing and Obstetrics, Celaya-Salvatierra Campus, University of Guanajuato, Mexico

**Keywords:** Motivation, Nutrition, University of Guanajuato

## Abstract

**Introduction:**

With high level of obesity in Mexico, there is a growing need to train more students in nutrition. Understanding what motivates students to choose pursuing degree in nutrition is very important for in post-secondary education. Better understanding of motivating factors may help educators to make sure that students complete their degress. The aim of this paper was to determine factors influencing student motivations for pursuing a bachelor's degree in nutrition at the University of Guanajuato.

**Methods:**

This was a survey study targeting 50 students at the University of Guanajuato. Questions in the survey targeted the following issues: general student characteristics, main reason for the choice of study area, objectives of higher education, university study field, and future plans. The descriptive statistics were calculated for the data obtained. The Z test was applied to analyze the differences between the ages.

**Results:**

The mean age of these students was 19 years; 74% were female while 26% were male. The students reported that the main reason for choosing a career in nutrition was personal preference and pointed out that such degree will give them better career options in the future, including better financial renumeration in comparison to other careers. Most of the students stated that their main reason for choosing University of Guanajuato was its prestige at the national and international level.

**Conclusions:**

Our study corroborates previously published study suggesting that students pursuing healthcare professions are motivaed by prestige and financial renumeration. With importance of nutrition in tackling obesity epidemic, it is very important to continue research on factors motivating students to choose careers in nutrition

